# Characterization of a murine model of monocrotaline pyrrole-induced acute lung injury

**DOI:** 10.1186/1471-2466-8-25

**Published:** 2008-12-17

**Authors:** Rio Dumitrascu, Silke Koebrich, Eva Dony, Norbert Weissmann, Rajkumar Savai, Soni S Pullamsetti, Hossein A Ghofrani, Arun Samidurai, Horst Traupe, Werner Seeger, Friedrich Grimminger, Ralph T Schermuly

**Affiliations:** 1 University of Giessen Lung Center (UGLC), Giessen, Germany; 2 Department of Neuroradiology, Justus-Liebig-University of Giessen, Germany; 3 Max-Planck-Institute for Heart and Lung Research, Bad Nauheim, Germany

## Abstract

**Background:**

New animal models of chronic pulmonary hypertension in mice are needed. The injection of monocrotaline is an established model of pulmonary hypertension in rats. The aim of this study was to establish a murine model of pulmonary hypertension by injection of the active metabolite, monocrotaline pyrrole.

**Methods:**

Survival studies, computed tomographic scanning, histology, bronchoalveolar lavage were performed, and arterial blood gases and hemodynamics were measured in animals which received an intravenous injection of different doses of monocrotaline pyrrole.

**Results:**

Monocrotaline pyrrole induced pulmonary hypertension in Sprague Dawley rats. When injected into mice, monocrotaline pyrrole induced dose-dependant mortality in C57Bl6/N and BALB/c mice (dose range 6–15 mg/kg bodyweight). At a dose of 10 mg/kg bodyweight, mice developed a typical early-phase acute lung injury, characterized by lung edema, neutrophil influx, hypoxemia and reduced lung compliance. In the late phase, monocrotaline pyrrole injection resulted in limited lung fibrosis and no obvious pulmonary hypertension.

**Conclusion:**

Monocrotaline and monocrotaline pyrrole pneumotoxicity substantially differs between the animal species.

## Background

Idiopathic pulmonary arterial hypertension (IPAH) is a severe disease characterized by elevated pulmonary blood pressure and pathological changes in the lung. These changes, such as endothelial injury, and the proliferation and migration of pulmonary vascular smooth muscle cells, lead to a reduction in the lumenal area of pulmonary vessels [1]. They are also accompanied by right ventricular hypertrophy and end-stage heart failure [2].

Animal models are important tools for the study of the pathogenic mechanisms of pulmonary hypertension (PH), and for the development of novel therapeutic strategies. Established models of PH include chronic exposure to hypoxia, and subcutaneous or intraperitoneal application of monocrotaline (MCT) or monocrotaline pyrrole (MCTP) in different mammals [3]. Increased shear stress by pneumonectomy [4], ductus arteriosus ligation [5,6] or anastomosis of the systemic arterial to the pulmonary arterial circulation [7,8] have been employed as alternative stimuli. Importantly, however, there are very few mouse models of PH, despite the obvious value of being able to manipulate the genetic background of mice. Chronic exposure of mice to hypoxia is the most common mouse model of PH. Under chronic hypoxic exposure, pulmonary vascular remodeling occurs within weeks. This remodeling process is characterized by *de novo *muscularization of previously non-muscular precapillary vessels, and right heart hypertrophy.

In transgenic mouse models, hypoxia has been repeatedly used to unmask the protective or deteriorating effects of a targeted gene. It has been shown that transient receptor potential channel 6 (TRPC6) knockout mice are protected against pulmonary vascular remodeling induced by hypoxia [9]. Similarly, 5-lipoxygenase knockout animals exhibit decreased vascular remodeling in response to hypoxia [10]. Conversely, prostacyclin receptor knockout mice [11] or mice over-expressing the 5-hydroxytryptamine transporter [12] develop more severe pulmonary hypertension. However, the nature of the remodeling differs between species, and it has been shown that mice kept under chronic hypoxia develop less PH than do rats maintained under comparable conditions [13].

The most aggressive form of PH in any animal model is induced by injection of MCT, which is extracted from plants of the genus *Crotalaria *[14,15], into rats. Although not completely understood, the proposed mechanism of action includes activation of the crude MCT alkaloid to the putative electrophile monocrotaline pyrrole (MCTP) [16,17] in the liver, which causes endothelial injury in the pulmonary vasculature with subsequent remodeling of the precapillary vessels and progressive pulmonary hypertension [18]. The limited opportunities to investigate genetic contributions to the pathophysiology of the pulmonary hypertension using this animal model highlight the importance of mice as laboratory animals. However, mice appear to be more resistant than rats to the effects of MCT, and these mice do not develop PH to the same degree. Injection or oral application of MCT to mice causes liver damage [19], modest pulmonary fibrosis [20-22] and immunotoxicity [23,24]. It has been proposed that liver metabolism of MCT to MCTP by the cytochrome P450 [25] system may differ between these species [25,26]. To circumvent this, *in vitro *methods have been developed to synthesize MCTP chemically [27], which is then stable in dimethyl formamide (DMF). Intravenous injection of the chemically-synthesized MCTP into the right atrium induces pulmonary hypertension in dogs [28,29].

The purpose of the present study was to investigate whether injection of MCTP results in PH in the two different mouse strains C57Bl/6 and Balb/c, and to characterize the course of the disease. Parts of this study were previously reported in an abstract to European Respiratory Society Conference 2006 [30].

## Methods

### Monocrotaline pyrrole synthesis

Monocrotaline pyrrole (MCTP) was chemically synthesized according using the methodology of Mattocks et al [27]. Briefly, 45 mg monocrotaline (Sigma-Aldrich, Germany) was diluted in 5 ml pure chloroform and mixed in a separation funnel with 45 mg *o*-chloranil (Fluka, Germany) in 5 ml chloroform. The solution was gently shaken and 0.5 ml of aqueous NaOH/NaBH_4 _(70:2; %:%) was added, and the mixture was vigorously shaken for 10 s, resulting in a blue biphasic solution. The clear, organic phase was separated and concentrated under vacuum. The resulting white crystals were weighed and solved in DMF.

### Thin layer chromatograms

Thin layer chromatograms were performed as described elsewhere [27]. Briefly, MCT or MCTP were run on silica-coated aluminium sheets (Merck, Germany) using light petroleum (b.p. 60–80°C):acetone (1:1) as eluting agent. Reaction with Ehrlich reagent results in a magenta color, which indicates the presence of MCTP, while reaction with *o*-chloranil followed by Ehrlich reagent detects both MCT and MCTP.

### Animals

Adult male C57Bl/6 or Balb/c mice (20–22 g body weight) and Sprague Dawley rats (300–350 g body weight) were obtained from Charles River Laboratories. Animals were housed in an environmentally-controlled animal facility for the duration of the experiment. Animals were provided with rodent chow and water *ad librium*. All experiments were performed according to the institutional guidelines that comply with national and international regulations.

#### Experimental protocol

Three groups, each consisting of five adult male Sprague Dawley rats randomly received s.c. injection of MCT (60 mg/kg) or i.v. injection of saline (0.5 ml) or MCTP (5 mg/kg). Hemodynamic measurements and assessment of right heart hypertrophy were made 28 days post MCT or MCTP injection.

To investigate the toxicity and survival rate, groups of eight C57Bl/6 and Balb/c mice received i.v. DMF (as vehicle) or MCTP at a dose of 6, 8, 10 and 15 mg/kg body weight. Hemodynamic measurements were made and right heart hypertrophy assessed after 28 days. Furthermore, seven groups, each consisting of 10 C57Bl/6 mice, received i.v. DMF or MCTP at a dose of 10 mg/kg body weight. Mice were randomly assessed for lung compliance, and cell counts in bronchoalveolar lavage fluid and histological changes in the lung were measured 3, 7, 14, 21 and 28 days after MCTP injection. Another group of five C57Bl/6 mice was i.v. injected with MCTP 10 mg/kg and subjected to flat-panel computed tomography at 3, 7, 14, 21 and 28 days.

#### MCT and MCTP injection

Injection of rats with the MCT was performed as described previously [31]. Control rats received subcutaneous saline. For MCTP injection, mice or rats were anesthetized by intra-peritoneal injection with ketamine/xylazine. During the procedure, animals were placed on a heating pad to maintain body temperature in the physiological range. The jugular vein was surgically exposed, and MCTP was injected intravenously according to the body weight using a low volume syringe (Hammilton, Swiss) and a 30 G needle. The incision was sutured and local disinfection was performed. Surgery was performed under aseptic conditions. After surgery, mice received oral antibiotics (Baytril^® ^1 ml/250 ml in drinking water).

#### Hemodynamic measurements

Hemodynamic measurements were performed in rats four weeks after MCT or MCTP administration, as described previously [31,32]. Mice were subjected to hemodynamic measurements 3, 7, 14, 21 and 28 days after MCTP injection. Animals were deeply anaesthetized as previously described [31,32], and the systemic arterial pressure (SAP) was monitored by cannulating the left carotid artery using a polyethylene cannula connected to a fluid-filled force transducer. The right jugular vein was used for catheterization of the right ventricle with a custom-made silicone catheter.

#### Lung compliance measurement

Lung compliance was measured as described previously [33]. Briefly, mice were anesthetized, tracheostomized and ventilated with 6 ml/kg body weight using a pressure-controlled ventilator.

#### Cell count in bronchoalveolar lavage fluid

After lung compliance measurements, BAL fluid (BALF) was sampled. Briefly, three successive instillations of 0.5 ml phosphate-buffered saline/EDTA (1%) were performed. The recovered BALF was centrifuged, the pellet was resuspended in saline solution and the total cell number was determined using a Neubauer chamber. For differential cell-counts, samples from BALF were spun onto a slide with a cytospin centrifuge (Shandon, Frankfurt, Germany) and stained with May-Grünwald-Giemsa. The relative numbers of each cell type were determined by direct assessment under 200× microscopic magnification, and expressed as a percentage of the total cell population present in the BALF.

#### Tissue processing

After measurement of hemodynamic parameters, animals were exsanguinated by opening the carotid artery and blood samples were collected. The left lobe of the lung was used to assess the lung wet/dry weight ratio measurement. The right lung was flushed through the pulmonary artery with saline, and fixed with a 2% formalin-based solution through the pulmonary circulation, using a constant pressure of 22 cm H_2_O. A counter pressure of 11 cm H_2_O was used on the alveolar side during fixation, in order to preserve the anatomical structure of the lung. Lung lobes were embedded in parafin blocks, and serial sections of 3 μm thickness were prepared and stained with Hematoxylin-Eosin and Masson's trichrome.

#### Flat-panel Computed Tomography

The fpvCT is a novel high-resolution CT system developed by General Electric (GE Global Research, Niskayuna, NY). In contrast to clinical CT scanners, an amorphous silicon flat-panel detector is irradiated by a cone-shaped X-ray beam. Animals were anesthetized by intraperitoneal injection of a mixture containing ketamine (Ketavet 100 mg/ml, Pharmacia & Upjohn) and xylazine (Rompun 2%, Bayer AG) and mounted on a table, which was then moved into the gantry bore during the scan, while the X-ray tube and detector, mounted on a rotating gantry, rotated around the table. The scan was performed in a sequential rather than a helical mode. For our investigations, 120 kVp at 40 mA was used. The scanning time for one rotation was 8 seconds, covering a field-of-view of 4.2 cm in the z-direction, sufficient for scanning the thorax of one mouse. Projection images were reconstructed using a cone-beam algorithm and an edge-defining reconstruction kernel [34,35]. Data can be reconstructed at arbitrary voxel sizes, but 0.05 mm^3 ^isotropic voxels were used for this investigation. All data were transferred to an Advantage Windows Workstation 4.1 (GE Health Care Europe, Buc, France) and processed with Volume Rendering software.

#### Data analysis

All data are given as the mean ± SEM. Differences between groups were assessed by ANOVA and Student-Newman-Keuls post hoc test for multiple comparisons, with a probability value < 0.05 regarded as significant.

## Results

### Analysis of MCTP and MCTP-injection in rats

**Figure 1:**
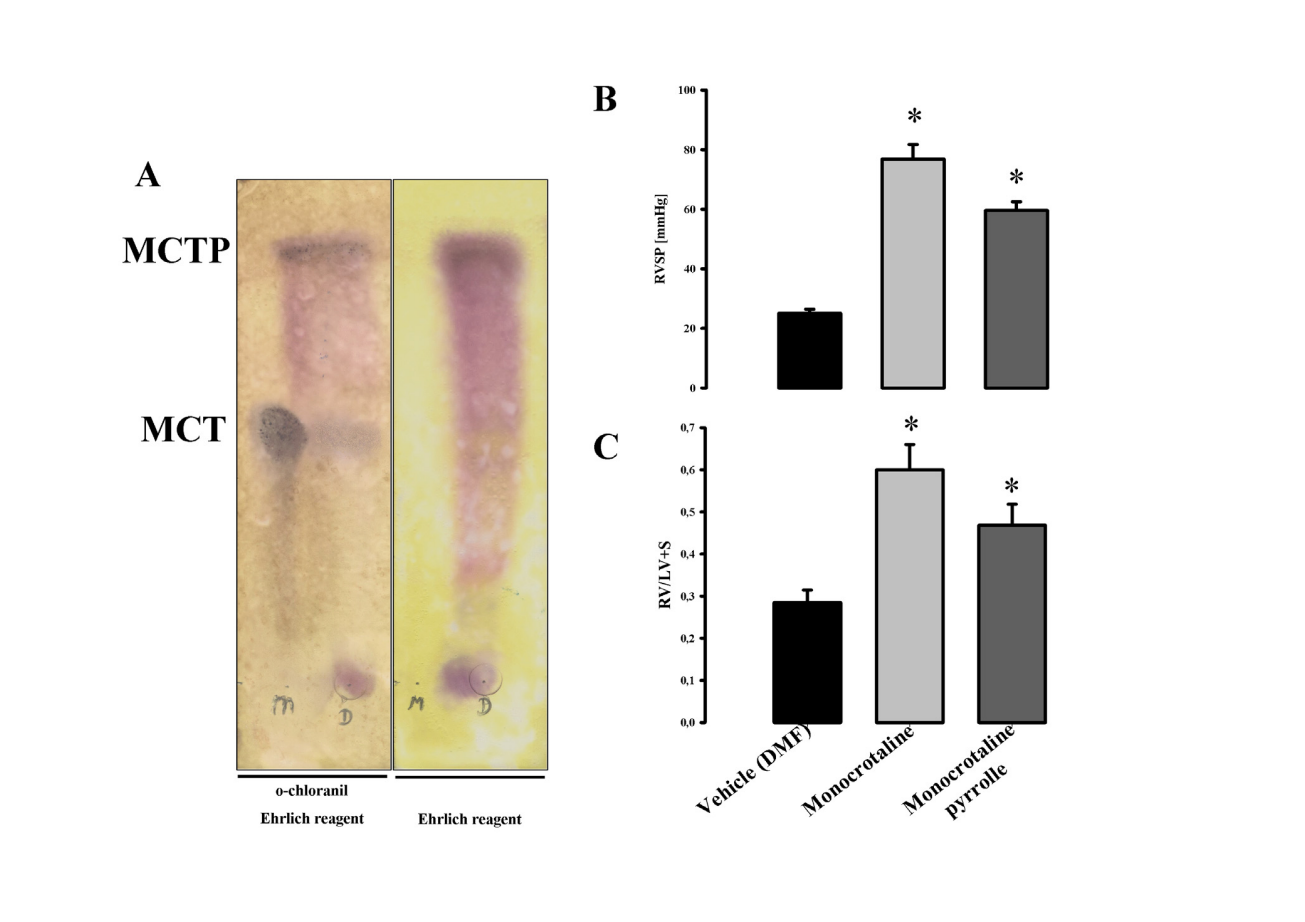
**Efficacy of monocrotaline pyrrole (MCTP) synthesis (A) and impact of MCTP and monocrotaline (MCT) on right heart hypertrophy (B) and pulmonary pressure (C) in Sprague Dawley rats**. Quality and purity of the final product MCTP were assessed by thin layer chromatograms (A). Rats were either subcutaneously injected with monocrotaline (60 mg/kg body weight) or intravenously with monocrotaline pyrrole (5 mg/kg body weight) and its vehicle DMF. Right ventricle pressure was measured four weeks after MCT or MCTP by right heart catheterization in anaesthetized animals. Right heart hypertrophy was assessed by calculating right ventricle/left ventricle plus septum (RV/LV+S) weight ratio. *, p < 0.05 versus DMF group.

The chemically-synthesized MCTP was subjected to thin layer chromatography (TLC) to evaluate the efficacy of chemical synthesis and MCTP purity. This revealed that the MCTP preparation obtained was pure (Figure 1A). Subcutaneous injection of MCT in Sprague Dawley rats at a dose of 60 mg/kg resulted in dramatic increase in right ventricular systolic pressure (76.87 ± 4.87 versus 25.08 ± 1.35 for control, *p < 0.05) and severe right heart hypertrophy (0.64 ± 0.01 versus 0.30 ± 0.01 for control, *p < 0.05) after four weeks. The MCTP was dissolved in DMF and injected intravenously into rats at a dose of 5 mg/kg. After four weeks, this led to elevated right ventricular systolic pressure (59.60 ± 2.93 versus 25.08 ± 1.35 for control, *p < 0.05, compared to controls) and significant right heart hypertrophy (0.47 ± 0.02 versus 0.30 ± 0.01, *p < 0.05, compared to controls). (Figure 1B and 1C). The effects induced by MCT and MCTP on right ventricular pressure and right heart hypertrophy were lower after MCTP injection than after MCT injection.

### Injection of MCTP in mice: mortality

**Figure 2:**
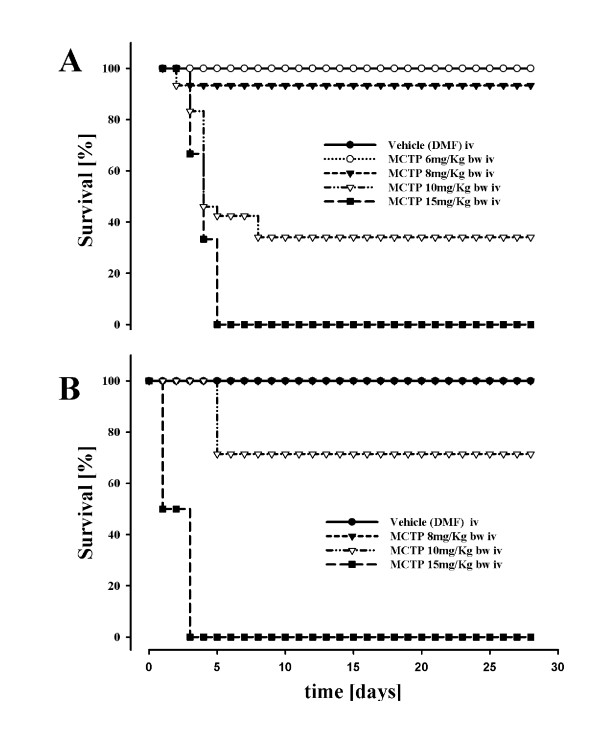
**Dose-dependant survival rate of mice treated with monocrotaline pyrrole**. C57Bl 6 (A) and Balb/c (B) mice were intravenously injected with different doses of monocrotaline pyrrole. Mice (n = 10 each group) were intravenously injected with monocrotaline pyrrole (MCTP) in doses varying from 6 to 15 mg per kilogram body weight or its vehicle (DMF). Survival was observed for the next 28 days after injection.

The MCTP was intravenously injected into C57Bl/6 mice at different doses. The MCTP had dose-dependent, toxic effects (Figure 2A). Similar effects were observed when mice with a Balb/c genetic background were injected with different doses of MCTP (Figure 2B). Interestingly, mortality was observed only between day 1 and day 4. The MCTP-injected animals exhibited decreased body weight, dyspnea, and developed lung edema and pronounced pleurisy. We further investigated in detail the lung changes in C57Bl/6 mice in the group treated with 10 mg/kg body weight, which exhibited a mortality rate of approximately 20%.

### Injection of MCTP in mice: histology and lung function

**Figure 3:**
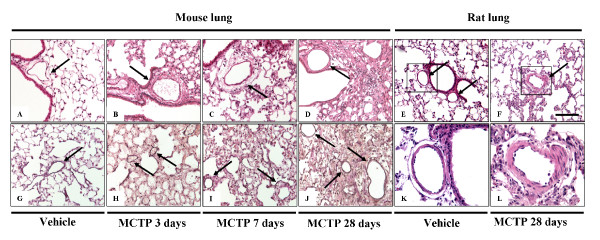
**Monocrotaline pyrrole-induced structural changes in lungs from C57Bl 6 mice and Sprague Dawley rats**. Representative histological pictures correspond to C57Bl 6 mice injected with DMF (vehicle) 28 days (A and G), MCTP 3 days (B and H), MCTP 7 days (C and I) and MCTP 28 days (D and J). Structural changes in rat lungs were assessed at 28 days after vehicle (E and K) or MCTP injection (F and L) Haematoxylin-Eosin staining is shown. Arrows indicate small pulmonary arteries. Scale bar = 50 μm.

**Figure 4:**
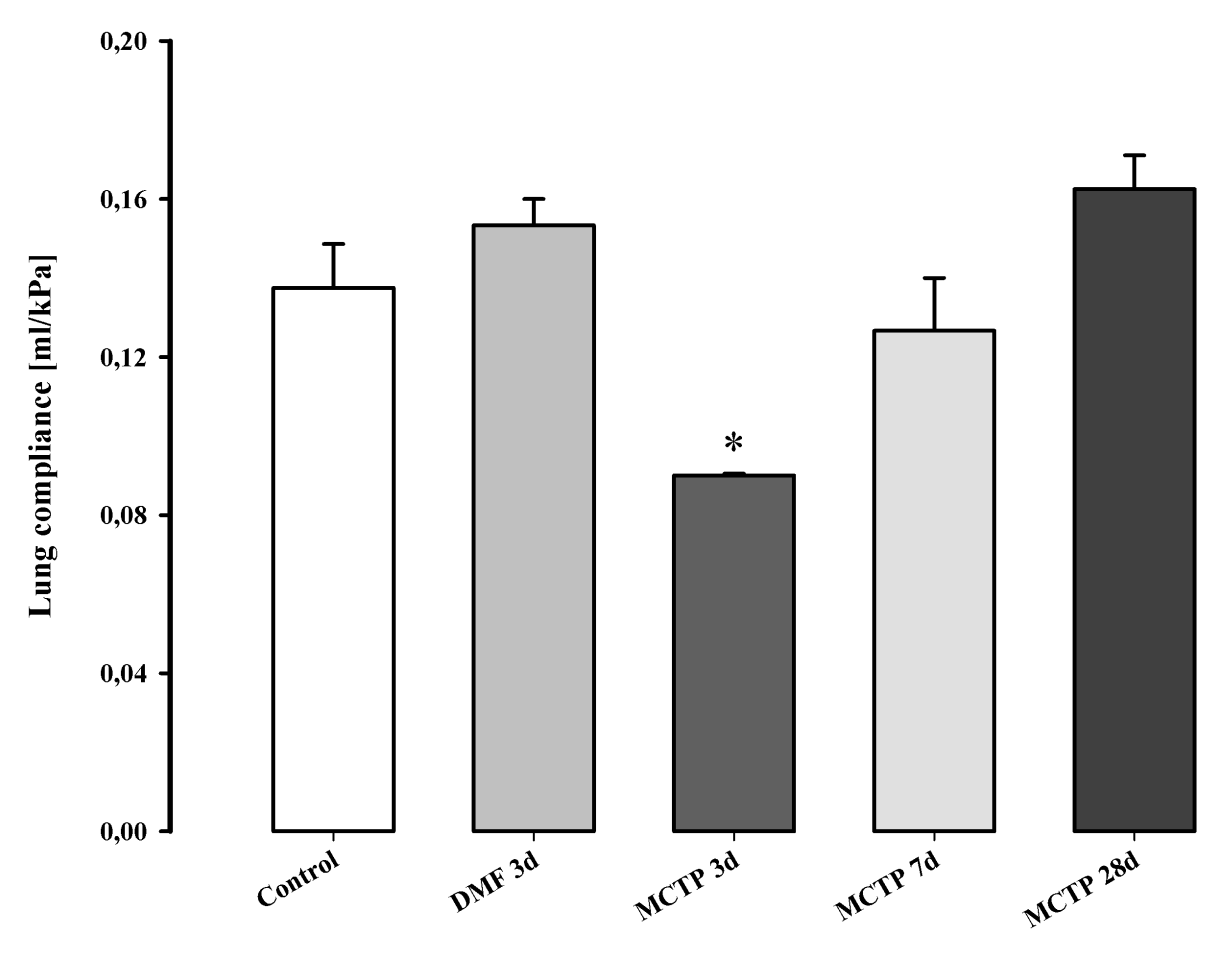
**Effect of monocrotaline pyrrole on lung compliance**. C57Bl 6 mice were intravenously injected with MCTP (10 mg/kg body weight) or vehicle. Lung compliance was measured at 3, 7 and 21 days. *, p < 0.05 versus control or DMF-injected animals.

**Figure 5:**
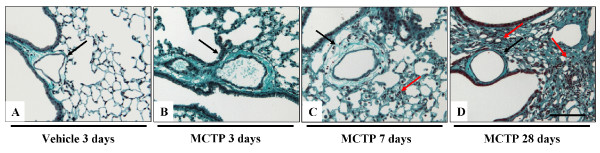
**Fibrotic changes in C57Bl 6 mice after monocrotaline pyrrole injection**. Mice received intravenous injection of MCTP 10 mg/kg body weight or DMF as vehicle. Masson's trichrome staining was performed on serial sections from Figure 3. Scale bar = 50 μm. Black arrows indicate small pulmonary arteries, red arrows indicate fibrotic changes.

**Figure 6:**
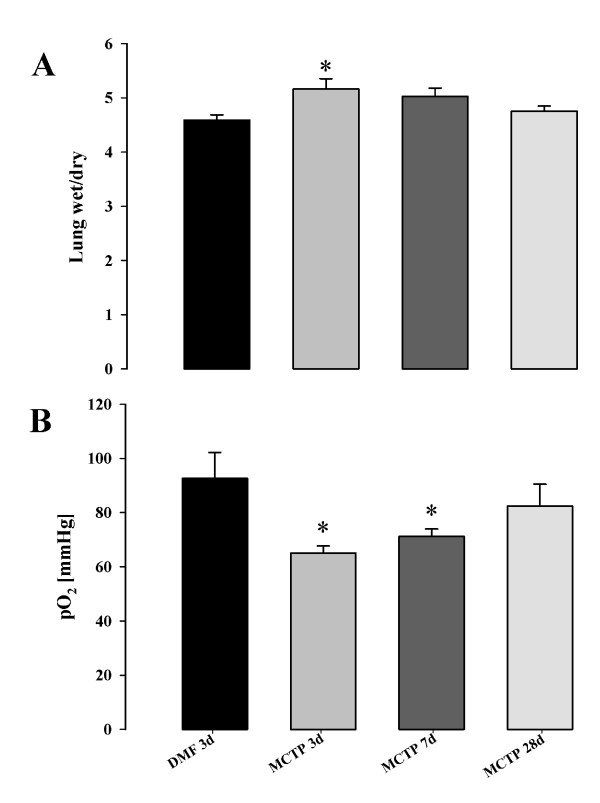
**Effect of monocrotaline pyrrole on arterial pO2 and lung edema**. Lung wet to dry weight ratio (A) and Arterial pO2 (B) were measured in C57Bl 6 mice at 3, 7 and 28 days after MCTP or vehicle (DMF) injection. *, p < 0.05 versus control or DMF-injected animals.

Hematoxylin-Eosin staining of the lungs from mice treated with 10 mg/kg body weight at different time points (Figure 3) in the early phase (three days after MCTP injection) revealed diffuse alveolar damage, alveolar exudates, interstitial edema with thickening of the alveolar septae, perivascular edema, and inflammatory cellular infiltrates. Lung edema was still present at seven days after treatment, but was resorbed by days 14–21. At day 28, no lung edema was apparent, but some areas of the lung developed fibrotic foci, starting on day 7 and culminating on day 28. These changes were accompanied by cellular inflammatory infiltrates, where lymphocytes were the prominent inflammatory cells present in the late phase, while granulocytes were prominent in the early phase. The vascular smooth muscle layer was not obviously affected at any of the time-points investigated, but consistent adventitial and perivascular edema was seen in at early-time points after MCTP injection (Figure 3). Fibrotic changes in mice injected with MCTP were assessed by Masson's trichrome staining and lung compliance measurements. In the early phase, lung compliance measurements revealed a dramatic drop (0.090 ± 0.0005 ml/kPa in MCTP-treated animals *versus *0.153 ± 0.0067 ml/kPa in vehicle-treated animals, p < 0.05) of the lung function (Figure 4) which, together with structural changes, indicated a massive reduction in the alveolar space and a reduction in parenchymal elasticity due to parenchymal and alveolar edema. In the late phase (28 days), lung compliance was similar to normal values, and lung edema disappeared, but localized limited fibrotic foci had formed. Interestingly, small pulmonary arteries did not show obvious signs of muscularization even in the fibrotic areas which were most likely the more strongly affected areas of the lung (Figure 5). Another assessment of lung edema is lung wet/dry weight ratio. This assessment revealed an increased ratio in the early phase for mice injected with MCTP in comparison to mice injected with vehicle (DMF) alone (Figure 6A).

### Injection of MCTP in mice: BAL fluid

**Figure 7:**
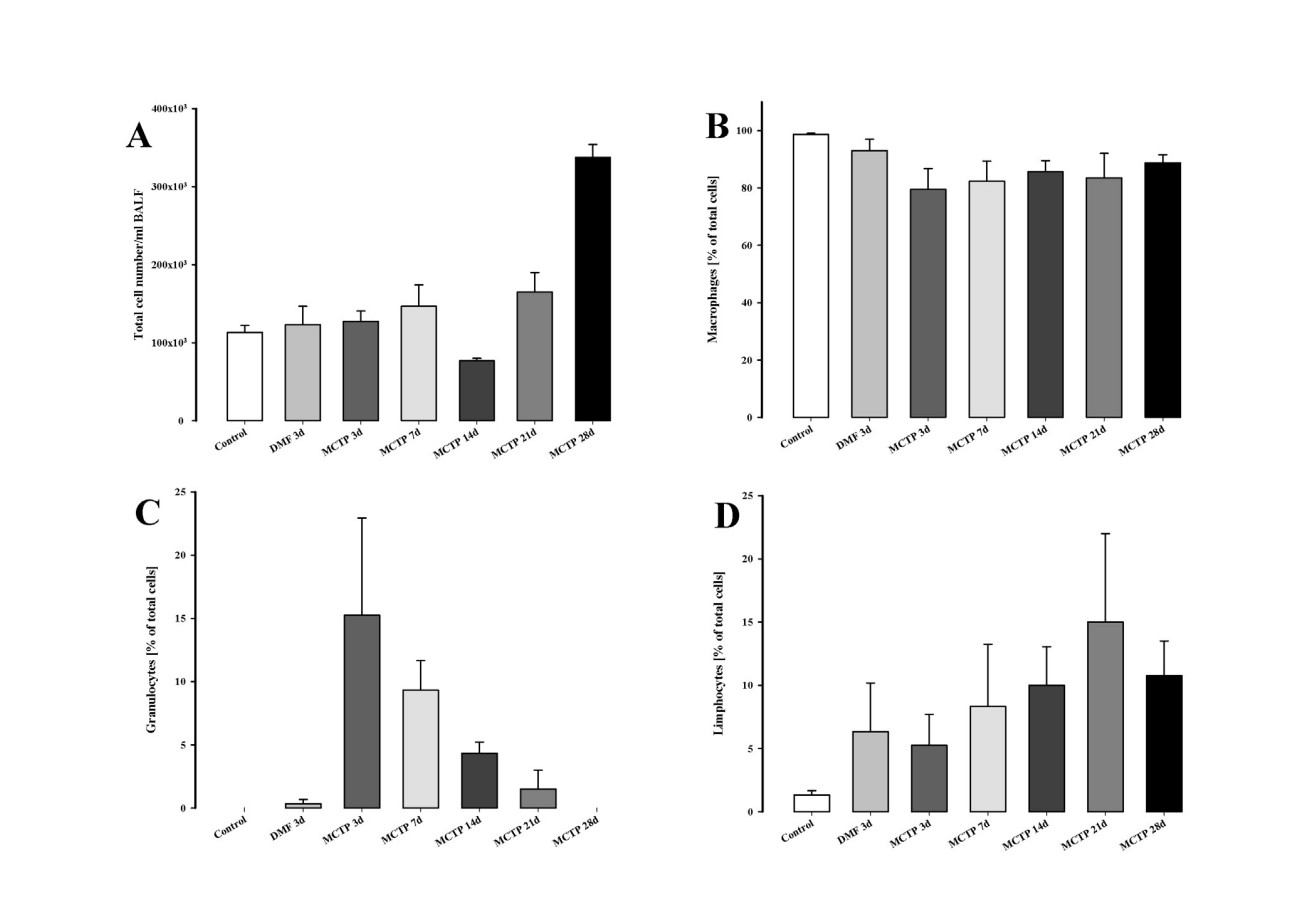
**Lung inflammatory changes in C57Bl 6 mice after monocrotaline pyrrole injection**. C57Bl 6 mice intravenously received MCTP (10 mg/kg body weight) or its vehicle (DMF). BALF was obtained after 3, 7, 14, 21 and 28 days. The inflammatory cellular profile in BALF was assessed as the total cell number (A) and the percentage of total cells of macrophages (B), granulocytes (C) and lymphocytes (D).

In order to confirm and investigate in greater detail the immune reaction to MCT administration, inflammatory cell numbers in BALF were assessed. Three days after MCTP injection, increased numbers of granulocytes (15.25 ± 7.68% of total cells in the MCTP-treated group *versus *0.33 ± 0.33% of total cells in the DMF-treated group) were present in BALF (Figure 7C), revealing acute lung inflammation. This phenomenon was reduced over time, up to day 28 (0.00 ± 0.00%) and, interestingly, was accompanied by an increased number of lymphocytes (10.75 ± 2.75% from total cells at day 28) (Figure 7D). Monocyte numbers did not change significantly over time after intravenous MCTP injection (Figure 7A, B, C and 7D).

### Injection of MCTP in mice: hemodynamics and gas exchange

Interestingly, gas exchange was affected by MCTP administration (Figure 6B). Arterial oxygenation (pO_2_) measured in blood collected from the carotid artery was significantly lower in MCTP-treated animals (65.06 ± 2.58) when compared to vehicle-treated animals (92.6 ± 9.6, p < 0.05)

**Figure 8:**
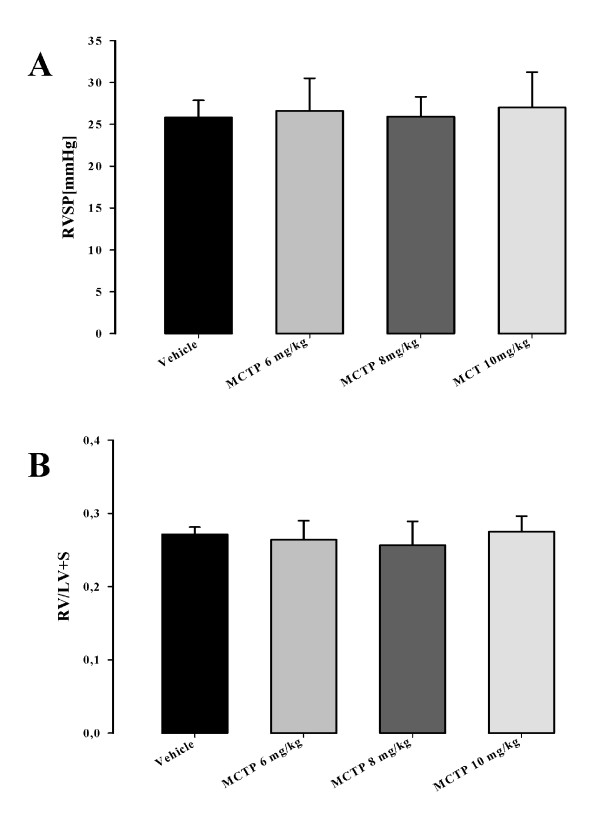
**Parameters of pulmonary hypertension in C57Bl 6 mice 28 days after injection with monocrotaline pyrrole in different doses**. Right Ventricular Systolic Pressure (A) and Right Ventricular Hypertrophy as the Futton index (B) is given.

**Figure 9:**
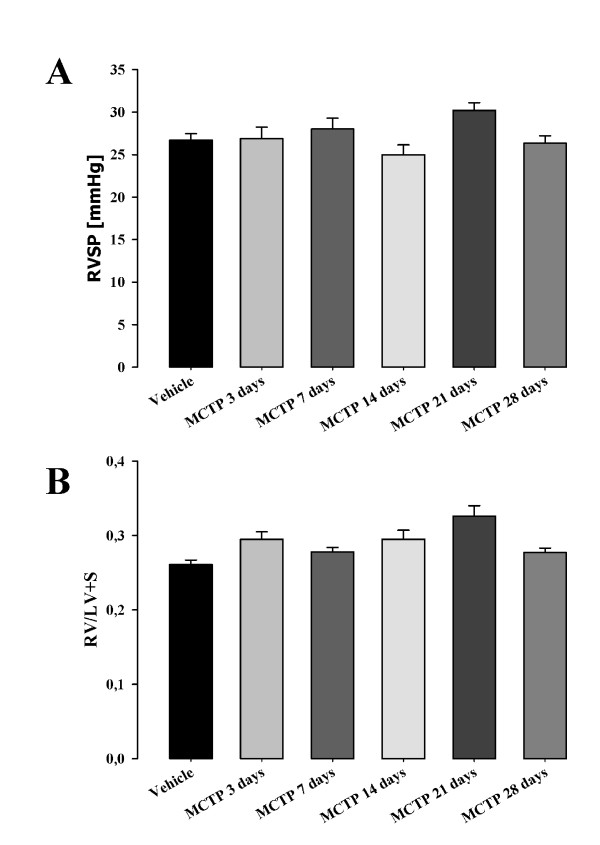
**Right ventricular systolic pressure (RVSP) (A) and right ventricular hypertrophy (RV/LV+S) (B) of C57Bl 6 mice injected with MCTP 10 mg/kg body weight**. Pulmonary hypertension parameters were measured at different time points: 3, 7, 14, 21 and 28 days.

When MCTP was intravenously injected into Sprague Dawley rats it induced elevated pulmonary arterial pressure and right heart hypertrophy, indicating pulmonary hypertension. We investigated whether the MCTP injury induces pulmonary hypertension when injected intravenously into mice. Right heart hypertrophy was assessed by right ventricle per left ventricle plus septum weight ratio (RV/(LV+S)). Before measuring RV/(LV+S), heart tissues were dried for one week at room temperature in order to avoid false-positive results due to heart edema. Pulmonary pressure was measured as right ventricular systolic pressure by right heart catheterization in anaesthetized animals. As depicted in Figure 8A, 28 days after MCTP injection, the pulmonary pressure did not vary significantly for animals injected with different MCTP doses 26.6 ± 3.9 (for 6 mg MCTP/kg), 25.9 ± 2.3 (for 8 mg MCTP/kg) and 27.0 ± 4.2 (for 10 mg MCTP/kg) *versus *animals injected with vehicle (25.8 ± 2.0 mmHg). These results are in line with the right heart hypertrophy assessments (Figure 8B) 0.264 ± 0.026 (for 6 mg MCTP/kg), 0.256 ± 0.032 (for 8 mg MCTP/kg) and 0.275 ± 0.021 (for 10 mg MCTP/kg) *versus *DMF-injected animals (0.271 ± 0.009). Pulmonary artery pressure was investigated at 3, 7, 14, 21 and 28 days after MCTP injection at a dose of 10 mg/kg body weight. Although a small elevation was observed in MCTP-treated animals (26.8 ± 1.3, 28.0 ± 1.2, 25.0 ± 1.1, 30.9 ± 0.9 and 26.3 ± 0.8 mmHg at 3, 7, 14, 21 and 28 days respectively), it did not differ significantly when compared with vehicle-treated animals (26.7 ± 0.7 mmHg) (Figure 9A). Similarly, small but not statistically significant changes in the RV/LV+S heart ratio were observed: 0.295 ± 0.010, 0.278 ± 0.006, 0.295 ± 0.012, 0.326 ± 0.014 and 0.277 ± 0.006 for 3, 7, 14, 21 and 28 days, respectively, after MCTP injection, *versus *0.261 ± 0.006 for DMF injected animals (Figure 9B).

### Injection of MCTP in mice: Computer tomography

**Figure 10:**
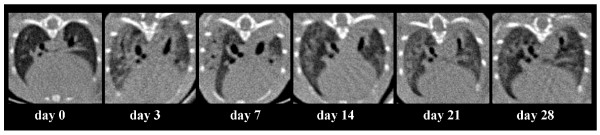
**Lung changes in C57Bl 6 mice injected with monocrotaline pyrrole at different time points assessed by Flat-panel Volumetric Computed Tomography (VCT)**.

In order to better understand and demonstrate the changes induced by intravenous MCTP injection into mice, mice were subjected to Flat-panel Volumetric Computer Tomography (VCT) before, and at different time points after, MCTP injection. As illustrated in Figure 10, in comparison with the initial lung structure, after MCTP injection the lung presented in the early phase increased, and exhibited a homogenous opacity indicating lung edema. This lung opacity diminished over time. In the late phase, at day 28, some heterogeneous lung opacities were still present, probably indicating fibrotic changes.

## Discussion

The aim of this study was to establish a new murine model of pulmonary hypertension by injection of monocrotaline (MCT), a plant alkaloid of *Crotalaria spectabilis*. In rats, the subcutaneous injection of MCT causes endothelial injury in the pulmonary vasculature with subsequent remodeling of the precapillary vessels (medial thickening, *de novo *muscularization of small pulmonary arterioles). Due to this mimicry of clinical pulmonary arterial hypertension, the rat MCT model has repeatedly been employed for investigating acute hemodynamic and gas exchange effects of vasodilators and the chronic anti-remodeling effects of anti-inflammatory and anti-proliferative agents [31,32,36]. Reproducible induction of severe, progressive pulmonary hypertension in response to MCT has been reported [37,38]. Although MCT has been used for the past five decades, the mechanism of action is still not clear. The proposed mechanism of action includes activation of MCT by liver cytochromes to a putative electrophile monocrotaline pyrrole (MCTP) which is highly reactive and possesses a short half life of 3–4 s in aqueous solution [39]. In the blood, it might circulate associated with red blood cells which stabilize and transport it to the active site, for example, the lung endothelium.

Previous studies have reported that the administration of MCT in mice in different forms, such as from the *Crotalaria spectabilis *plant [40], by the oral administration of crude alkaloid [21,23,26] or subcutaneous injections [20,22,41,42]. Vascular and lung parenchymal inflammation, lung edema, moderate lung fibrosis and minor right heart hypertrophy were reported in these studies. Most of these studies have suggested that mice are comparatively deficient in the liver enzymes that convert MCT to its active metabolites [20,25,41].

As mice are highly attractive for pulmonary hypertension research due to the possibility of using genetically-engineered animals, multiple groups have attempted to establish new mouse models of pulmonary hypertension [21,22,41,42]. We chemically dehydrated MCT to MCTP according to a methods of Mattocks [27] and injected different doses intravenously into two different mouse strains (C57Bl 6 and BALB/c). Thin layer chromatograms confirmed the purity of chemically-synthesized MCTP. In addition, a positive control experiment included the injection of the synthesized MCTP into rats. This confirms MCTP as being the active metabolite of MCT, and demonstrates the efficacy of chemical synthesis. The difference in the degree of pulmonary hypertension induced by MCTP and MCT might correlate with the different bioavailability, and dose differences (5 mg/kg for MCTP versus 60 mg/kg for MCT). When injected intravenously, MCTP led to dose-dependant mortality of both mouse strains within the first week due to dyspnoea, pleural liquid accumulation, lung edema and severe infiltration of inflammatory cells into lung interstitium. Those animals which survived exhibited similar signs and symptoms including minor elevation of pulmonary pressure and right heart hypertrophy which was not significant compared to the control animals. A slight increase in pulmonary artery pressure was present in the early phase after MCTP administration and was not sustained or progressive. This phenomenon may be attributed to massive lung injury and inflammation which is usually associated with local cytokine and other mediator release, for example, of TNF or thromboxane [43,44]. In this regard, Molteni *et al *reported dysregulation of angiotensin converting enzyme, plasminogen activator and prostacyclin (PGI_2_) after MCT injection in mice [21]. Histopathological changes in the lungs of mice injected with MCTP consisted in the early phase of perivascular and interstitial lung edema and diffuse alveolar damage with alveolar hyaline membranes and inflammatory cell infiltration. The cellular profile in the bronchoalveolar lavage fluid demonstrated the significant increase in granulocytes which is a characteristic feature of acute respiratory distress syndrome [45,46]. The abovementioned histopathological changes, together with the inflammatory syndrome, are hallmarks of acute lung injury. The initial inflammation and alveolar damage was followed in the later phase by moderate localized lung fibrosis. Lung compliance decreased significantly only in the early time points due to fluid accumulation. Interestingly, these animals seem to recover over time in terms of lung compliance, exhibiting similar values at 28 days to the control animals. Histopathological changes assessed by Haematoxylin-Eosin and Masson's trichrome staining revealed obvious fibrotic changes.

Similar changes have been reported by previous studies in which MCT was administered in different forms. A major objective of this study was to investigate whether MCTP induces pulmonary hypertension in mice. We carefully investigated the physiological and histological parameters of pulmonary hypertension in these animals. Pathologically, these animals presented with a pseudo-wall-thickness of small pulmonary arteries induced by adventitial and perivascular edema, but there was no obvious sign of muscularization or neo-muscularization of these vessels. Right ventricular systolic pressure measured by right heart catheterization in anaesthetized animals 28 days after MCTP injection was slightly elevated. However, this did not reach statistically significant levels in any of the groups injected with different doses. This observation is consistent with the assessment of right ventricular hypertrophy and histopathological changes reported by Hayashi et al [22]. In fact, small pulmonary capillary hypertension is one of the physiological abnormalities of the acute respiratory distress syndrome [47]. Recently, Raoul *et al*. reported elevated pulmonary pressure and right heart hypertrophy in mice 15 days after MCTP injection into the tail vein [48]. Although we used an appropriate and even double the dose of MCTP reported in that study, our study did not reveal significant differences in pulmonary hypertension-related parameters which may be dependant on the mouse strain or the MCTP synthesis. However, considering the importance of a mouse model of pulmonary hypertension, numerous research groups will experiment with different technical approaches which might prove successful. Our findings demonstrate that interspecies differences in response to MCT and lung diseases are not limited to the liver metabolism of these alkaloids. Future experiments assessing mouse and rat endothelial cells in response to MCTP might reveal the basis of the interspecies variability. A technical limitation of the study might be related directly to the MCTP application: a bolus injection of MCTP *versus *slow release after subcutaneous MCT injection. However, bolus injection of MCTP in rats results in pulmonary changes which consistently differ from those induced in mice.

## Conclusion

To conclude, this study reports pathological pulmonary changes induced by an application of the putative electrophile MCTP in mice. Administration of MCTP in mice and rats results in acute lung injury. In rats, but not in mice, it ultimately leads to severe and progressive pulmonary hypertension. To our knowledge, this is the first study to report and characterize an animal model of acute lung injury induced by monocrotaline pyrrole in mice. Thus, MCTP injection in mice may be a valuable animal model to investigate both the efficacy of new therapies for acute respiratory failure and fibrotic repair mechanisms.

## Competing interests

The authors declare that they have no competing interests.

## Authors' contributions

RD, SK, AS and ED performed animal experiments. AS performed TLC. RD and RTS drafted the manuscript RS, SP and HT carried out imaging investigations. NW performed statistical analysis. HAG, HT, WS and FG participated in the sequence alignment and in the design of the study. RTS conceived the study and participated in its coordination.

## Pre-publication history

The pre-publication history for this paper can be accessed here:


